# Complex Multiplanar Fracture of the Distal Femur with an Undescribed Pattern: A Case Report and Review of the Literature

**DOI:** 10.7759/cureus.7533

**Published:** 2020-04-04

**Authors:** Deepak Kumar, Lokesh SN, Praveen Sodavarapu, Shahnawaz Khan, Pratik M Rathod

**Affiliations:** 1 Orthopaedics, Post Graduate Institute of Medical Education and Research, Chandigarh, IND; 2 Orthopaedics, Employees' State Insurance Corporation Hospital, Chennai, IND

**Keywords:** distal femur fracture, trauma, complex distal femur fracture, fracture fixation, intraarticular distal femur fracture

## Abstract

Distal femur fractures in younger age groups are associated with high-impact injury leading to severe comminution and soft tissue injuries. Most of the intra-articular distal femur fractures occur as a result of axial loading accompanied by a variable amount of flexion. An 18-year-old male patient who had met with a road traffic accident was brought to the trauma center. Radiographic examination revealed a fracture of the distal femur, predominantly involving the lateral condyle without any evidence of metaphyseal comminution, and lateral view showed a complete separation of both the condyles from the proximal shaft. This type of fracture pattern did not fit into any of the current classification systems of distal femoral fractures. The medial and lateral approaches were carried out simultaneously instead of the anterior midline approach, owing to the poor skin condition over the anterior aspect of the knee. These fractures are difficult to treat due to high articular involvement and present a tedious task for the surgeon. Anatomical reduction with preserved articular cartilage is the key to a good outcome in such complex fractures. Atypical fracture types are not uncommon, and they can be incorporated into existing or future classification systems, which may contribute to a better understanding and management of these fractures.

## Introduction

Distal femur fractures account for about 3-4% of femoral fractures and 0.4% of all fractures [1]. Young men and older women are commonly affected either due to high-velocity injuries or domestic accidents. Dynamic muscular and static-loading forces constantly act on the distal femur [2]. The aim of this case report is to document an unusual fracture pattern of the distal femur, which could not be classified based on any of the existing classification systems. We observe that a dual incision approach is warranted to fix such complex distal femur fractures in some cases. We also want to emphasize that all fractures cannot be incorporated into the present-day classification system and that we need further diversification into new types.

## Case presentation

**Figure 1:**
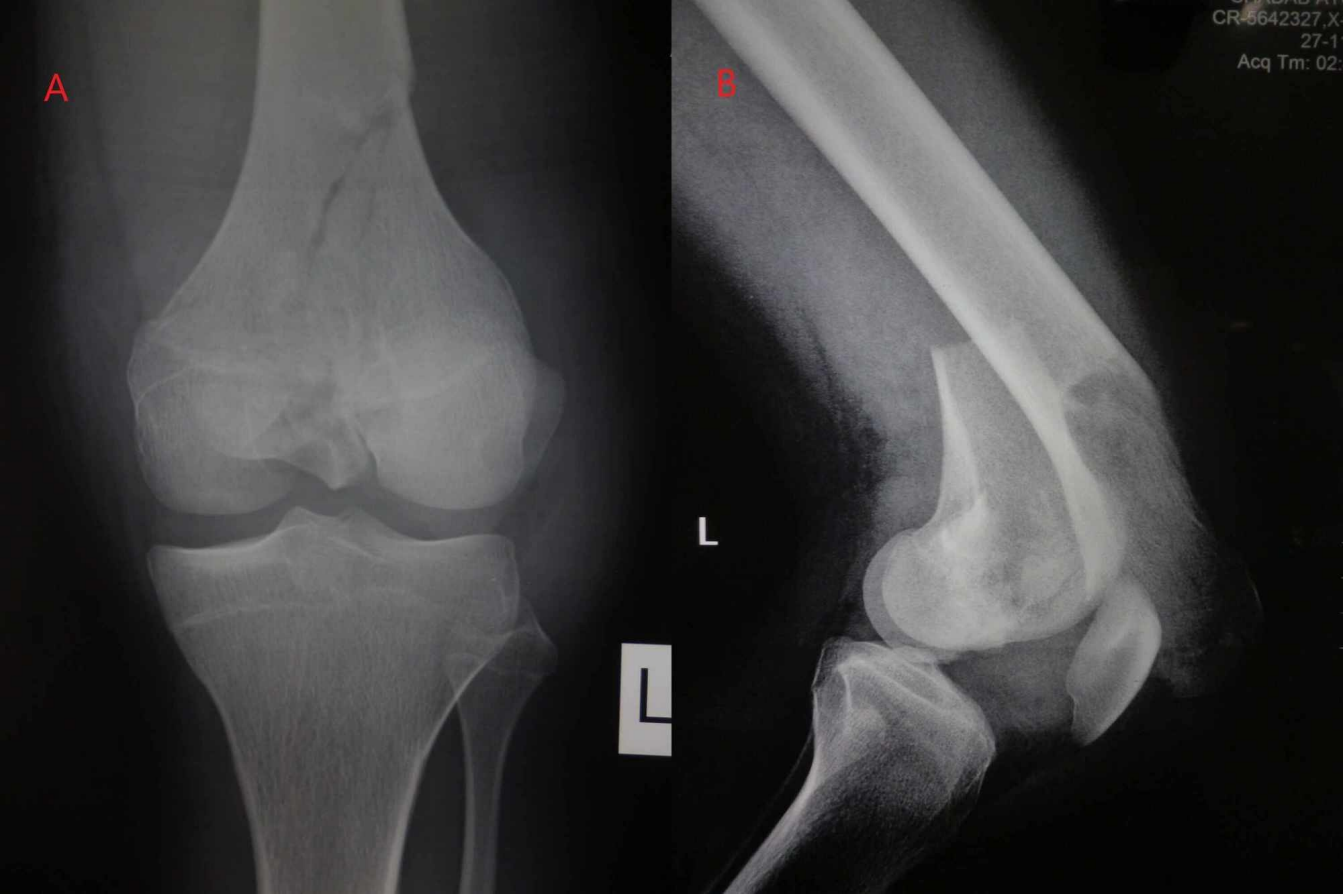
Preoperative radiographs of the knee A: anteroposterior view; B: lateral view. The images show distal femur fracture

An 18-year-old male patient who had met with a road traffic accident was brought to our emergency. He had been hit by a truck while riding on his motorcycle. On evaluation, the patient complained of pain and inability to move the left lower limb. There was swelling over the left distal femur and knee joint, and tenderness was elicited on palpation. There was a superficial abrasion over the anterior aspect suggestive of direct impact over the knee. The distal neurology and vascularity were intact. Radiographic examination revealed a fracture of the distal femur, predominantly involving the lateral condyle without any evidence of metaphyseal comminution, and lateral view showed complete separation of both the condyles from the proximal shaft (Figure 1).

**Figure 2:**
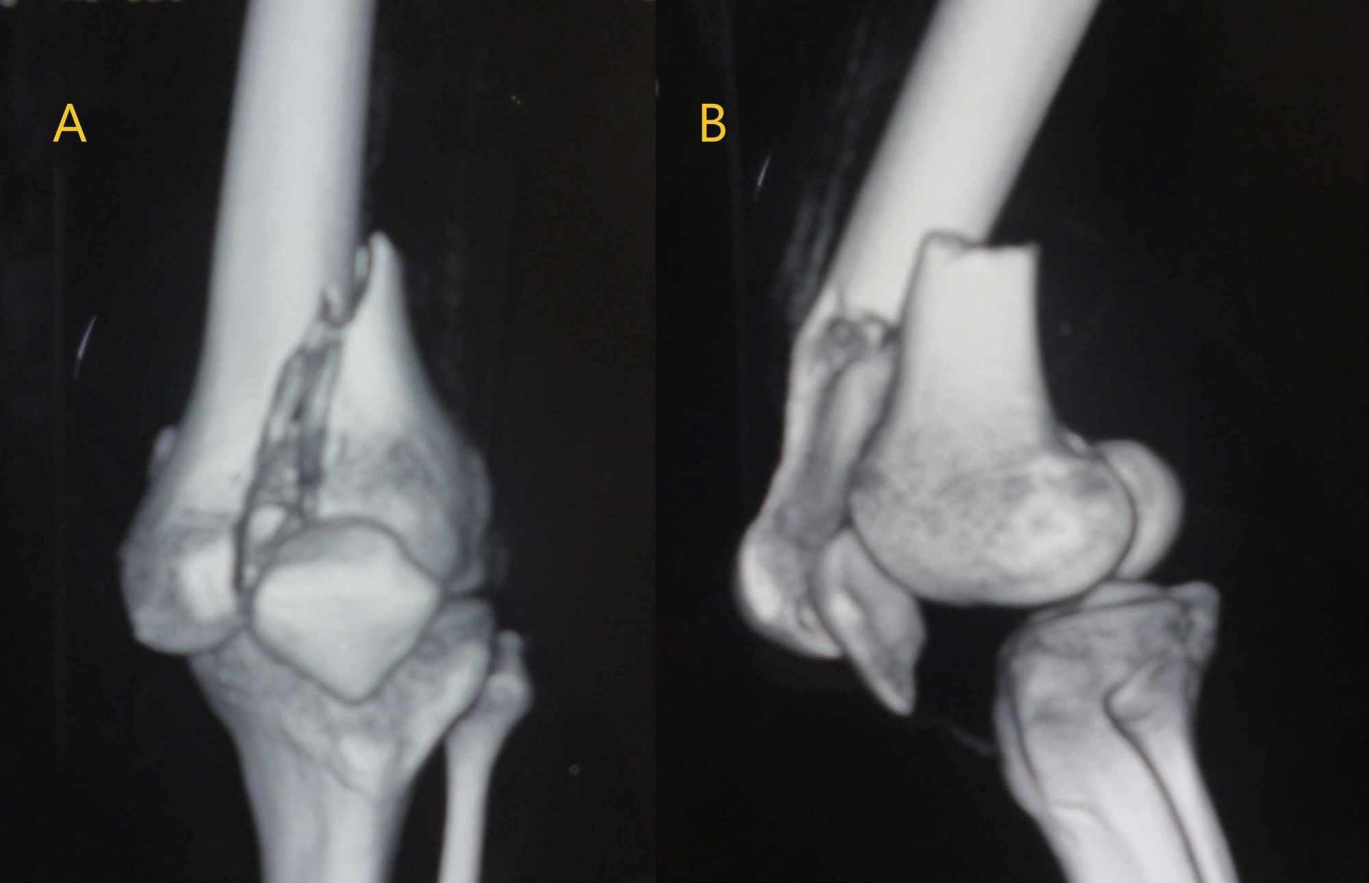
Three-dimensional reconstruction of the CT scan A: anteroposterior view; B: lateral view. The images show the sagittal intercondylar separation of the distal femur and the proximal shaft with part of the articular surface (coronal fracture) in continuation with the intact metaphysis CT: computed tomography

A CT scan revealed an unusually complex intraarticular fracture of the distal femur, which did not fit into any of the classification systems used currently. The fracture pattern consisted of sagittal intercondylar separation of distal femur without metaphyseal comminution, and contrary to previously described fracture patterns, the proximal fragment (femoral shaft) had some part of the articular surface (coronal fracture) in continuation with intact metaphysis, which was lying anterior to the patella. This type of fracture pattern did not fit into any of the classification systems currently used for distal femoral fractures (Figure 2).

**Figure 3:**
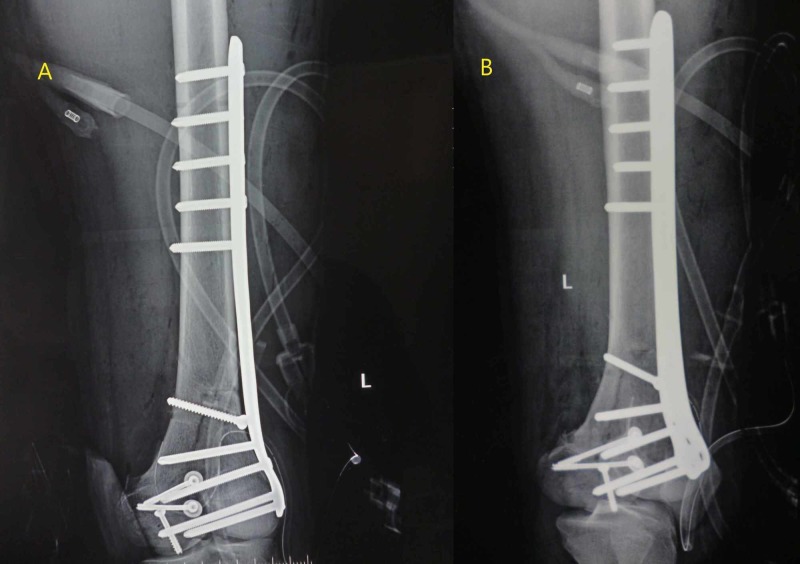
Immediate postoperative radiographs of the knee A: anteroposterior view; B: oblique view. The images show the reduction of the fracture with distal femur locking plate and screws

The patient was shifted to the operation theatre. Medial and lateral approaches were carried out simultaneously instead of the anterior midline approach, owing to the poor skin condition over the anterior aspect of the knee. The medial condyle was reduced first and stabilized with multiple Kirschner wires (K-wires), followed by the fixation of two medial condylar fragments with small-fragment, partially threaded cancellous screws. After the medial condyle reconstruction, the lateral condyle was reduced and temporarily stabilized with K-wires, followed by definitive fixation with a lateral distal femoral locking plate (Figure 3).

**Figure 4:**
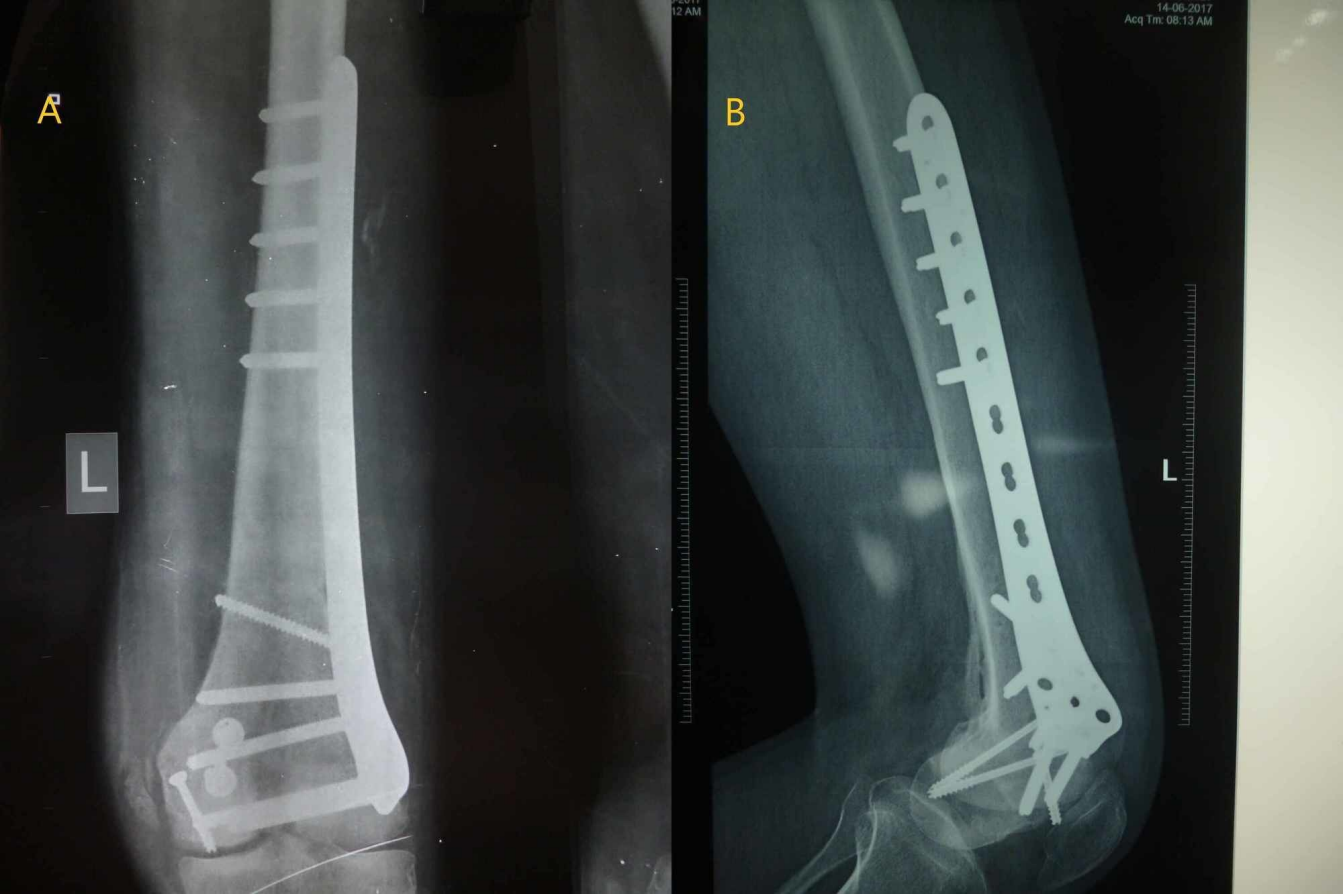
Radiographs of the knee at the final follow-up A: anteroposterior view; B: lateral view. The images show complete bony union

**Figure 5:**
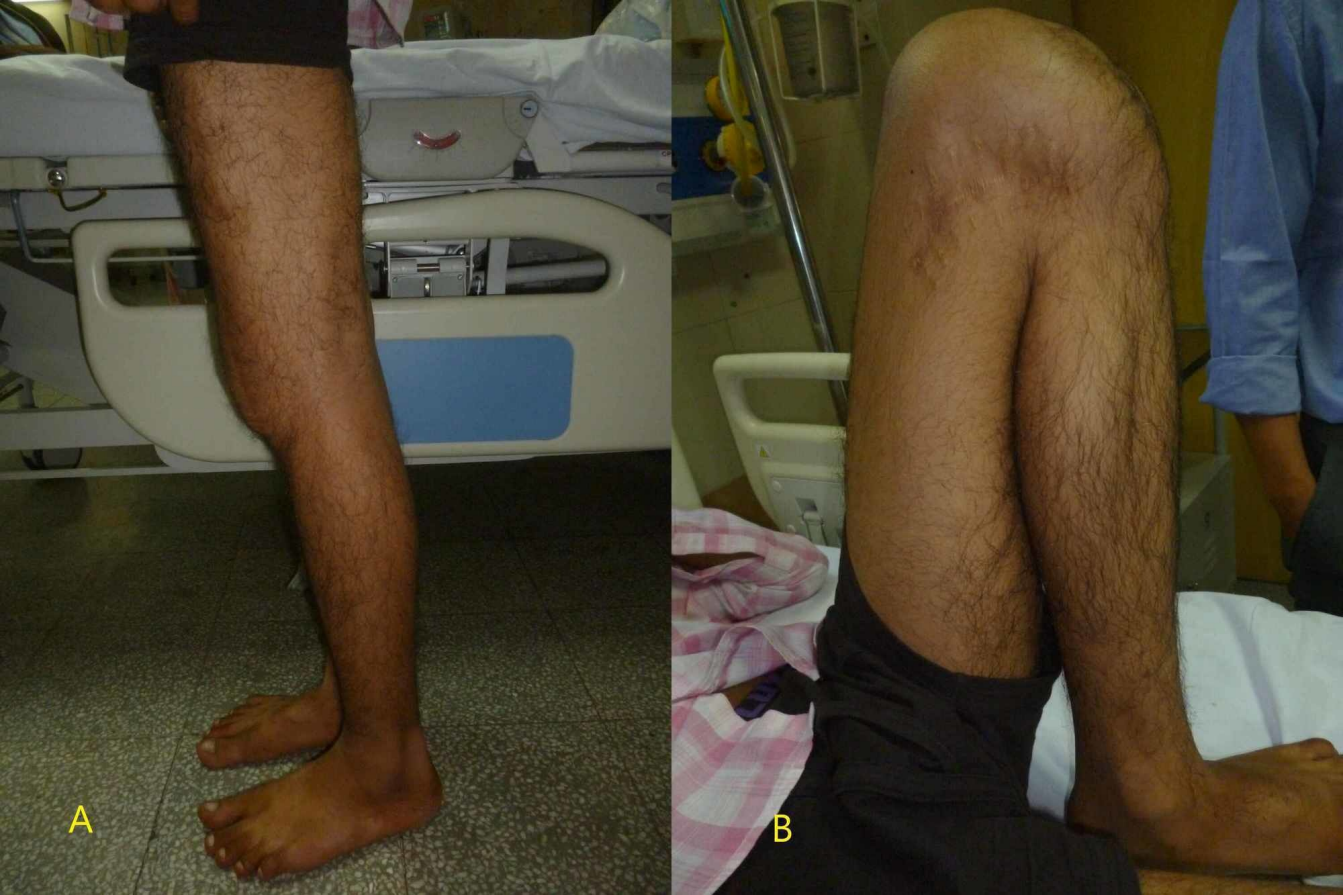
Clinical images of the knee at the final follow-up A: image showing complete extension; B: image showing complete flexion

The postoperative period was uneventful. The wound healed, and the sutures were removed. The patient was kept on an aggressive physiotherapy protocol, and knee range of motion (ROM) and isometric quadriceps were started on the second day post-surgery. A continuous passive motion (CPM) machine was employed to start a passive movement of the knee joint, and by the end of the first week, 90 degrees of passive motion was achieved while the active motion was restricted due to pain. After two weeks, a good range of active flexion of 0-90 degrees was achieved by the patient. Weight-bearing was started with toe touching with the aid of crutches in the first week after surgery and progressively moved to partial weight-bearing in the third week, followed by full weight-bearing at six weeks. Full knee ROM of 0-120 degrees of flexion was achieved. The complete radiological and clinical union was achieved by 12 weeks post-surgery. At the final follow-up after 2.5 years post-surgery, the radiographs showed complete bony union, and there was full ROM equal to opposite normal limb, and the patient was completely pain-free [International Knee Documentation Committee (IKDC) subjective knee score after two years: 86/100]. There was no evidence of arthritis in the knee joint, and good alignment was maintained. The patient was able to sit cross-legged and squat at the final follow-up and did not have any functional disability. The patient had issues of occasional mild swelling of the knee joint but without any functional disturbance. The patient has attained pre-injury activity levels without any difficulty (Figures 4, 5).

Proposed mechanism of injury

**Figure 6:**
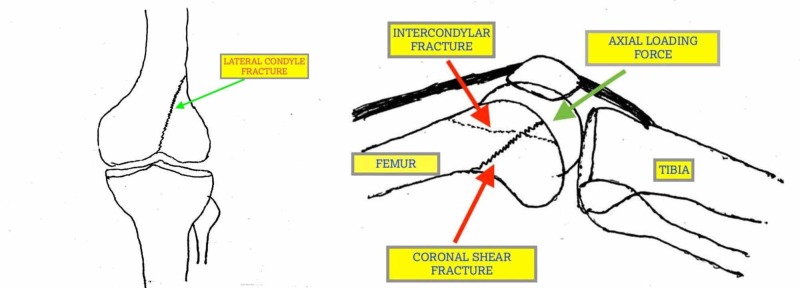
Schematic illustration of the fracture with proposed mechanism of injury

Most of the intraarticular distal femur fractures occur as a result of axial loading accompanied by a variable amount of flexion resulting in different fracture patterns. In this particular injury, with the knee in flexion and patella engaged in the intercondylar groove, the initial impact was on the patella. This created a fracture of the intercondylar region and simultaneous further flexion of the knee and continued axial loading, resulting in the coronal shear injury of the medial condyle of the femur. Further displacement of fragments occurred due to the strong pull of quadriceps, hamstrings, and gastrocnemius (Figure 6).

## Discussion

Distal femur fractures have a bimodal distribution and are very uncommon, accounting for only 0.4% of all the fractures [1]. These fractures are difficult to treat due to high articular involvement and pose a tedious task for the surgeon. The common complications are non-union, malunion, pain, decreased ROM arising from articular incongruity, and inadequate fixation [3]. In our literature search, we could not find any classification system into which our fracture pattern could be incorporated or any article describing the pattern which we have discussed above. A total of three classification systems have been mainly proposed for distal femur fractures: 1) Neer et al. (1967), 2) Seinsheimer classification (1980), and 3) the Müller AO Classification (1990) [4-6].

Neer et al. proposed one of the simplest and earliest classification systems, and they identified three categories of fracture patterns [4]. However, this system had some disadvantages: it did not include coronal plane fractures, nor did it prognosticate the outcomes. Little clinical information was available to the surgeons; consequently, this system has fallen out of favor and is seldom used nowadays, with only historical importance accorded to it.

Seinsheimmer divided the distal femur fractures into four types [5]. He had found that type 1 and 2 fractures were commonly seen in osteoporotic fractures and involved low energy trauma. Type 4 fractures involved high energy trauma. This system also failed to gain popularity as it was not user-friendly and provided minimal information on prognosis.

The AO system was introduced by Muller in 1990 [6]. This was found much easier to use and satisfied all criteria for an ideal classification. Muller described three basic groups: type A fractures - extra-articular fractures; type B fractures - partial articular fractures, where parts of the articular surface remain in contact with the diaphysis of the femur; and type C fractures - complete articular fractures with both condyles being detached from the diaphysis. These were subdivided depending on comminution and displacement. Type B fractures included Bl (sagittal, lateral condyle), B2 (sagittal, medial condyle), and B3 (frontal, Hoffa type). Fracture type C was further divided into C1 (articular simple, metaphyseal simple), C2 (articular simple, metaphyseal multi-fragmentary), and C3 (multi-fragmentary). This classification system prognosticated that as fractures progress from type A to C, the severity of injury increases and the prognosis of a good outcome decreases. This system has been universally accepted as the gold standard of fracture classification.

Distal femur fractures in the younger age group are associated with high-impact injury leading to severe comminution and soft tissue injuries, which further result in poor functional outcomes [3]. There is sparse literature on classification systems for distal femur fractures. Our case presented management challenges owing to the unclassified pattern of the fracture and the issue of poor soft tissue anteriorly over the knee. Due to the above-mentioned difficulties, we gave dual incision for exposure, and an atypical fracture configuration warranted multiple screw placement in different directions.

A variety of surgical exposures, reduction techniques, and various new implants have been developed to treat such complex fractures. Recognition of these injuries is important for preoperative planning and good outcomes. Routine radiographs may not be of sufficient value in determining the extent and type of the fracture pattern. In such cases, oblique-view radiographs and CT scans are recommended as they are more efficient in determining the fracture patterns, which allows for more rigorous preoperative planning.

The assessment of ligamentous injuries along with distal femur fractures is also of equal importance, for they play a crucial role in postoperative physiotherapy, rehabilitation, and functional outcomes of the knee. The intraoperative assessment of our case had shown no injury to the collateral or cruciate ligaments, which also contributed to a rapid recovery and good functional outcome in our patient. Anatomical reduction with preserved articular cartilage is the key to a good outcome in such complex fractures.

## Conclusions

Distal femur fractures occur due to high-velocity injuries in young patients, while the geriatric population is susceptible to these fractures even with low-velocity injuries. Compromised soft tissue envelope and fracture comminution make the management of these fractures difficult. Timely surgical intervention and adherence to the principles of intra-articular fracture fixation can lead to good functional outcomes in the treatment of this condition. Atypical fracture types are not uncommon, and they can be incorporated into existing or future classification systems, which may contribute to a better understanding and management of these fractures.
